# New Treatment of Diphtheria

**Published:** 1881-04

**Authors:** 


### A NEW TREATMENT OF DIPHTHERIA.

There are various modes of treatment, some of them directly opposed to one another; but all of them have repeatedly failed. Any method which seems reasonable or plausible is worth trying, and what purports to be a new discovery appears to be worthy of consideration. A young man in the West, whose arm had been amputated, was recently attacked with the disease before the limb had healed. To the surprise of his physician, the matter incident to diphtheria appeared on the arm where it had been severed, in place of depositing itself, as usual, in the throat, and the case proved to be a very mild one. The doctor profited by this strong intimation from nature, and when next called to visit a diphtheritic patient, blistered his chest. There most of the deposits showed themselves, and the patient speedily recovered. Hence, it is inferred that the disorder generally affects the throat on account of the thinness of its lining, and not because it is any part of its morbid law to do so. When the blister breaks the epidermis, the tenderness of that portion of the body draws the virus in the system thither, instead of to the throat, as ordinarily. This may prove to be of vast advantage to the medical fraternity. If it should, it would be only one of many instances in which what we call accident has revealed more than any amount of science.

### TWO HARMLESS DOSES THAT MAKE ONE POISON.

Chlorate of potassium and iodide of potassium are both entirely harmless in suitable doses. Furthermore, these two salts do not react upon each other in solution, even at a boiling heat. Yet it has been proved that, when they are administered together, they do combine in the stomach, producing iodate of potassium, which is poisonous M. Melsens found that dogs could take the chlorate or iodide in doses of from 5 to 7 grammes with impunity, but that a mixture of the two killed them in a few days, with the symptoms of poisoning by iodate of potassium. This combination must, therefore, be avoided. Indeed, as a general rule, the chlorate is so unstable, and so ready to give up its oxygen, that it cannot safely be combined with any substance capable of oxidation.—*American Journal of Pharmacy.*

Vennor's open winter has been so open that a lot of snow escaped.

